# Evaluation of 3-dimensional rotational venography for the diagnosis of non-thrombotic iliac venous lesion

**DOI:** 10.3389/fcvm.2023.1088224

**Published:** 2023-02-03

**Authors:** Yingjiang Xu, Jian Wu, Yongjia Cheng, Gang Chen, Xinqiang Han, Yuguo Sheng, Xuejun Wu, Wenming Wang

**Affiliations:** ^1^Department of Interventional Vascular Surgery, Binzhou Medical University Hospital, Binzhou, Shandong, China; ^2^Department of Vascular Surgery, Shandong Provincial Hospital, Cheeloo College of Medicine, Shandong University, Jinan, Shandong, China

**Keywords:** non-thrombotic iliac vein lesions, 3D rotational venography, 2D-digital subtraction angiography, diagnosis, iliac vein

## Abstract

**Background:**

The purpose of this study was to determine the technical feasibility and safety of 3D rotational venography (3D-RV) in the diagnosis of non-thrombotic iliac vein lesions compared with traditional 2D-digital subtraction angiography (2-DSA).

**Methods:**

The general epidemiological data (including age, gender), clinical manifestations (including major symptom, affected extremity, CEAP classification, comorbidity, stenosis rate), and intra-operative findings (iliac vein indentation position, collateral circulation, procedure time, X-rays dose, contrast agent dosage) of 61 NIVL patients who were assessed by 3D-RV and traditional 2-DSA between October 2018 to October 2022 were obtained and analyzed.

**Results:**

A total of 61 consecutive patients with symptomatic NIVL from our institution were enrolled in this study. With the aggravation of iliac vein stenosis, the proportion of indicators such as contralateral formation and iliac vein compression indentation reflecting the severity of compression under 3D-RV reconstruction increased significantly. Also, significant differences were observed between the 3D-RV and 2-DSA groups concerning procedure time (10.56 ± 0.09 s vs. 12.59 ± 0.37 s; *p* < 0.01), X-ray dose (41.25 ± 0.21 mGy vs. 81.59 ± 1.69 mGy; *p* < 0.01) and contrast agent dosage (21.48 ± 0.24 mL vs. 33.69 ± 0.72 mL; *p* < 0.01). Contralateral iliac vein imaging (*p* = 0.002), pelvic collateral vein imaging (*p* = 0.03), and external iliac vein indentation (*p* = 0.001) were found to influence the severity of iliac vein compression.

**Conclusion:**

3D-RV can display dynamic stereo image information of NIVL, augmenting the information obtained from traditional 2-DSA. Contralateral iliac vein imaging, pelvic collateral vein imaging, and external iliac vein indentation can be used to evaluate the severity of iliac vein compression to some extent.

## Introduction

Along with advancements in angiography, clinicians have gradually realized that venous disorder and their recurrence are related to iliac vein compression, resulting in proximal venous outflow obstruction (PVOO) (1). One of the primary mechanisms of PVOO is iliac vein obstruction (IVO), which may be thrombotic or compressive (2). Although there is no criterion standard for identifying patients who require treatment of non-thrombotic iliac vein lesions (NIVL), stenting of the iliac vein has become increasingly recognized as a treatment for PVOO, with reports citing significant improvement in pain, swelling, ulceration, and stasis dermatitis. Intravascular ultrasound (IVUS) is the gold standard method for diagnosing and treating NIVL but is only available at a few medical centers in China (3, 4). Thus, traditional 2D-digital subtraction angiography (2-DSA) is the main imaging method for evaluating iliac vein disease, however two-dimensional images have some limitations, such as underestimating the stenosis lesions and the inability to evaluate the anatomical structure around the iliac vein (5).

Cone beam computed tomography (CBCT) is a well-established imaging modality that has recently been integrated into the C-arm angiography suite (6). CBCT is based on the rotational movement of the C-arm equipped with a flat-panel detector around the patient, and the acquired images are post-processed to form a 3D image for endovascular procedures and image-guided therapy (7, 8). 3D rotational venography (3D-RV) has now become mainstream in the evaluation of cerebrovascular diseases, and it can be performed on flat-panel fluoroscopy equipment using 1 or 2 rotational acquisitions, each lasting 4–5 s (9, 10). This study aimed to evaluate the 3D-RV in diagnosing NIVL compared to traditional 2-DSA.

## Methods

### Patients

This study was approved by the hospital research ethics committee. The inclusion criteria were: (1) the lower extremity deep vein anterograde angiography by dorsal foot venipuncture demonstrated iliac vein compression, which was preliminarily screened as an iliac vein compression lesion. Combined with lesion and normal vein, the diagnosis of NIVL was more than a 50% area of stenosis of the iliac vein (11). After NIVL diagnosis, intervention was considered if requesting by the patient. (2) the clinical, present, etiology, anatomy, and pathophysiology (CEAP) grade was C3-C6. The exclusion criteria were: (1) a history of serious trauma or major surgery within the last 4 weeks; (2) pregnancy; (3) uncontrollable hypertension (systolic blood pressure >180 mmHg or diastolic blood pressure >110 mmHg); (4) history of a cerebral hemorrhage in the last 3 months; (5) life expectancy < 1 year; (6) contraindications to iodinated contrast media; (7) severe heart, liver, and kidney insufficiency; (8) a history of deep-vein thrombosis. The general epidemiological data (including age, gender), clinical manifestations (including major symptom, affected extremity, CEAP classification, comorbidity, stenosis rate), and intra-operative findings (iliac vein indentation position, collateral circulation, procedure time, X-rays dose, contrast agent dosage) of 61 NIVL patients who were assessed by 3D-RV and traditional 2-DSA between October 2018 to October 2022 were obtained and analyzed.

### Image technique capture

The images were captured using a GE IGS530 DSA machine, GE AW4.7 post-processing station, MEDRAD Incorporated Mark 07 Series high-pressure syringes (Medrad, USA), and contrast agent iodixanol injection (320 mg/mL, Hengrui Pharmaceuticals Co., Ltd. Jiangsu, China).

**Figure 1:**
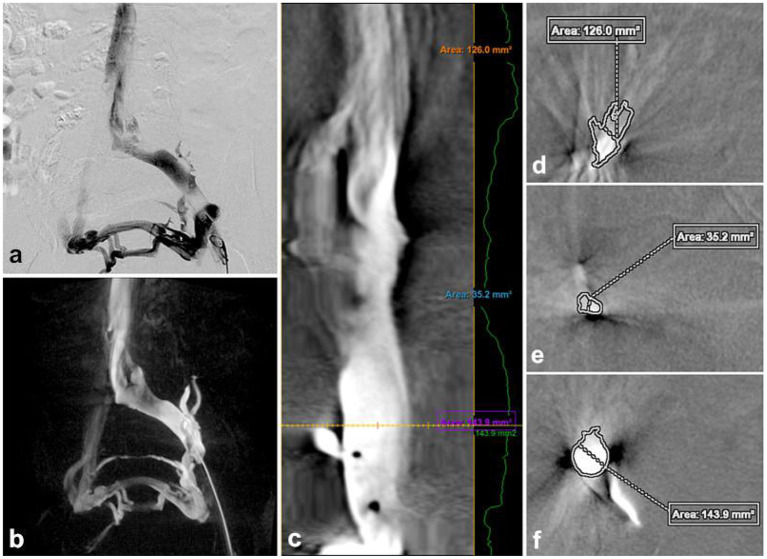
Traditional DSA image **(a)**, 3D-RV image **(b)**, the vascular was straightened **(c)** to measure the normal area of the proximal **(d)** and distal of the stenosis **(f)**, the cross-sectional area at the narrowest point **(e)**. Stenosis rate = [1 – (2Se/Sd+Sf)] × 100% = [1–2 × 35.2/126 + 143.9] 100% = 73.92%.

Traditional 2-DSA images were captured with the patient in a supine position. The Seldinger technique was used to access the femoral vein by introducing a 6F sheath (Terumo Corporation, Tokyo, Japan) under local anesthesia. Under X-ray guidance, a 5F pigtail catheter (Terumo Corporation, Tokyo, Japan) was positioned in the external iliac vein. The contrast agent iodixanol (diluted to a concentration of 185 mg/150 mL) was injected with a high-pressure syringe (600 psi, an injection rate of 7 mL/s, and an injection volume of 15 mL). Anteroposterior (AP), oblique and lateral projection venography images were obtained while the patient was holding their breath (Figure 1a).

3D-RV images were captured by placing a 5F pigtail catheter in the external iliac vein for venography to determine the lesion anatomy. The C-arm fluoroscopy mode was set to 3D-CT, with a contrast agent injection rate of 4 mL/s, a total volume of 8 mL, a pressure of 300 psi, and delayed exposure of 0.5 s. The 3D-RV was performed with an appropriate rotation degree (200°, 40°/s), a ray delay time of 1.5 s, a contrast agent injection rate of 4 mL/s, a total volume of 24 mL, and a pressure of 300 psi. Acquired images were sent to a 3D workstation, and 3D-RV was computationally reconstructed using Innova 3D sub software. The image was post-processed to visualize the critical parameters (Figure 1b).

### Observed parameters and calculation methods

The critical parameters included the iliac vein stenosis rate, collateral veins, iliac vein indentation location, radiation dosage, procedure time, and contrast agent dose. On the 2-DSA lateral view, the minimum distance between the anterior and posterior (AP) walls of the most compressed portion of the iliac vein was recorded as distance a, with the AP calibers of the iliac vein proximal and distal to the compressed area recorded as distance b and distance c, respectively. The iliac vein stenosis rate was calculated as [1 – 2a^2^/ (b^2^ + c^2^)] × 100%. In the 3D-RV images, the proximal to the distal end of the stenosis lesion was connected to form a central line, and the “Two Click AVA” shortcut key was used to straighten the vessels and measure the normal and minimum compressed cross-sectional area of the vessels (Figure 1c). The percentage stenosis area of the potential venous compression point was calculated as [1 – (area of compressed cross-section/area of normal cross-section)] × 100% (Figures 1d–f). Based on the iliac vein stenosis rate under 2-DSA, the severity of iliac vein compression was divided into group A (stenosis rate 50–65%), group B (stenosis rate 66–80%), group C (stenosis rate >80%) to further evaluate the effect of 3D-RV on iliac vein compression with different degrees of stenosis. The etiology of the iliac vein stenosis was evaluated by identifying extrinsic and intrinsic details of the iliac vein using multiplanar 3D-RV images. The iliac collateral veins were observed in detail, especially distal to the lesion area, to assist in diagnosis and treatment. The radiation dosage and procedure time of each 2-DSA and 3D-RV procedure were recorded based on the fluoroscopy machine reports. Each contrast dose was recorded in detail by the imaging technician.

### Statistical analysis

All statistical analysis was performed using SPSS statistics version 26 (SPSS Inc., Chicago, IL, USA). Continuous variables are presented as median and range values in the case of a non-parametric distribution, and comparisons were made using the Mann-Whitney test. Continuous variables are presented as the mean and standard deviation in the case of a parametric distribution, and comparisons were made using the independent *t*-test. Categorical variables were compared with the chi-square test and Fisher's exact test, and they are reported as frequencies and percentages. The significance of collateral veins imaging and iliac vein indentation location for the diagnosis of NIVL was assessed by logistic regression analysis. A value of *p* < 0.05 was considered statistically significant.

## Results

A total of 61 NIVL patients with endovascular management were involved in this study, of which 43 (70.49%, 43/61) were women, and their median age was 58 years old (ranging from 35 to 87). The major symptoms associated with symptomatic NIVL included pain (59.02%) and swelling (68.85%). Among the 61 patients, 14 (22.95%) had hypertension, 21 (19.67%) had diabetes mellitus, and 10 (16.39%) had hypercholesterolemia. Moreover, 24.95% (15/61) had a C3 classification lesion, 34.43% (21/61) had a C4 classification lesion, 22.95% (14/61) had a C5 classification lesion, and 18.03% (11/61) had a C6 classification lesion.

**Figure 2:**
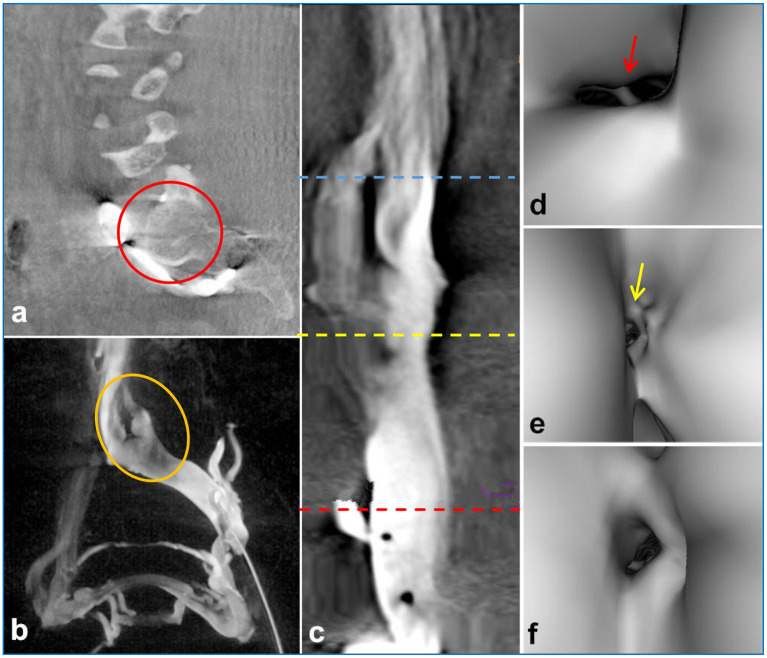
3D-RV image depicted compression of the iliac vein by the posterior lumbar spine [**(a)**, red circle]. The coronal view showed an indentation of the left common iliac vein [**(b)**, yellow circle]. Intravascular virtual endoscopic imaging displayed the entrance of the inferior vena cava [**(c)** blue line, **(d)** arrows], stenosis [**(c)** yellow line, **(e)** arrows] and normal intraluminal morphology [**(c)** red line, **(f)** arrows], and the “ridge” morphology at the compression site of the left common iliac vein [**(d)**, arrows].

3D-RV revealed that the main cause of NIVL was compression of the iliac vein by the right common iliac artery, with the axial view revealing pathological changes in the lumbar spine, such as disc herniation, osteophytes, and spondylolisthesis (Figures 2a, b). Moreover, vascular virtual endoscopic imaging revealed a “ridge” in the lumen (Figures 2c–f). Spinal degeneration was common in patients with senility and occurred in 11 of 61 NIVL patients, including local osteophytes (6.56%; 4/61), simple lumbar disc herniation (4.92%; 3/61), intervertebral disc herniation and local osteophyte formation (4.92%; 3/61), lumbar spondylolisthesis and spondylolisthesis, osteophyte formation (1.64%; 1/61). Lumbar pathological changes could significantly decrease the space between the lumbar spine and the right common iliac artery aggravating the stenosis of the left common iliac vein. The clinical features and imaging findings of the patients are summarized in Table 1.

**Table 1:** Clinical features and imaging findings of non-thrombotic iliac vein lesions.

**Variable**	**Total, *n* = 61**
Age, years (range)	58 (35,87)
Gender, female, *n* (%)	43 (70.49)
**Major symptoms**	
Pain, *n* (%)	36 (59.02)
Swelling, *n* (%)	42 (68.85)
Affected extremity, left, *n* (%)	53 (86.89)
**CEAP classification**	
C3, *n* (%)	15 (24.59)
C4, *n* (%)	21 (34.43)
C5, *n* (%)	14 (22.95)
C6, *n* (%)	11 (18.03)
**Comorbidity**	
Hypertension, *n* (%)	14 (22.95)
Diabetes mellitus, *n* (%)	12 (19.67)
Hypercholesterolemia, *n* (%)	10 (16.39)
**Etiological diagnosis**	
Spinal degeneration	11 (18.03)
Intraluminal ridge	6 (9.84)

With the aggravation of iliac vein stenosis, the proportion of indicators such as contralateral formation and iliac vein compression indentation reflecting the severity of compression under 3D-RV reconstruction increased significantly. The imaging effect of 3D-RV on iliac vein compression in each subgroup is illustrated in Table 2. The stenosis rate was observed in 77.83 ± 1.33 vs. 74.5 ± 1.405% (*p* = 0.67) of patients. Significant differences were observed between the 3D-RV and 2-DSA groups concerning procedure time (10.56 ± 0.09 s vs. 12.59 ± 0.37 s; *p* < 0.01), X-ray dose (41.25 ± 0.21 mGy vs. 81.59 ± 1.69 mGy; *p* < 0.01) and contrast agent dosage (21.48 ± 0.24 mL vs. 33.69 ± 0.72 mL; *p* < 0.01) (Table 3). The degree of iliac vein stenosis was used as the dependent variable (50–65% was defined as mild, 66–80% was defined as moderate, >80%was defined as severe, and assign values 1, 2, and 3, respectively). Collateral veins imaging (pelvic collateral veins, other collateral veins, contralateral iliac veins, with development assigned “1,” without development assigned “0”) and iliac vein indentation location (IVC junction, common iliac vein, external iliac vein, with indentation assigned “1,” without indentation assigned “0”) were used as independent variables for ordered categorical logistic regression analysis. Contralateral iliac vein imaging (*p* = 0.002), pelvic collateral vein imaging (*p* = 0.03), and external iliac vein indentation (*p* = 0.001) were found to influence the severity of iliac vein compression. The imaging features of 3D-RV iliac vein stenosis analyzed by multi-classification logistic regression are displayed in Table 4.

**Table 2:** The imaging effect of 3D-RV on iliac vein compression per subgroup.

**3D-RV**		**50–65%** **(*n* = 14,%)**	**66–80%** **(*n* = 19,%)**	**>80%** **(*n* = 28,%)**
**Collateral veins imaging**	Pelvic collateral veins	9 (64.29)	13 (68.42)	21 (75)
	Contralateral iliac veins	6 (42.86)	10 (52.63)	18 (64.29)
	Other collateral veins	3 (21.43)	4 (21.05)	5 (17.86)
	Common iliac vein	4 (28.57)	9 (47.37)	19 (67.85)
**Iliac vein indentation location**	External iliac vein	2 (14.29)	10 (52.63)	17 (60.71)
	IVC junction	12 (85.71)	13 (68.42)	18 (64.29)

**Table 3:** Comparison of key parameters during the procedure.

**Variable**	**3D-RV**	**2-DSA**	***p*-value**
Stenosis rate (%)	77.83 ± 1.33	74.5 ± 1.405	0.67
Procedure time, s	10.56 ± 0.09	12.59 ± 0.37	< 0.01
X-rays dose, mGy	41.25 ± 0.21	81.59 ± 1.69	< 0.01
Contrast agent dosage, mL	21.48 ± 0.24	33.69 ± 0.72	< 0.01

**Table 4:** Multiclassification logistic regression analysis of 3D-RV iliac vein stenosis imaging findings.

**Imaging features**	**Wald χ^2^**	**OR**	**95% CI**	***p-*value**
Pelvic collateral veins	9.08	3.88	1.86–4.76	0.03
Contralateral iliac veins	11.41	3.27	0.96–3.49	0.002
Other collateral veins	0.43	−0.69	−2.21–0.53	0.76
Common iliac vein	4.42	0.93	0.77–4.49	0.08
External iliac vein	16.79	4.62	1.94–4.01	0.001
IVC junction	0.81	0.26	0.56–2.82	1.05

## Discussion

Accurate diagnosis of NIVL is a prerequisite for effective treatment. The etiology of NIVL is mainly related to anatomical factors (12). Long-term compression of the iliac vein leads to blockage of venous return, which increases the occurrence of acute lower limb deep vein thrombosis and even pulmonary embolism (13). 2-DSA is insufficient in NIVL condition assessment, stent placement decision-making, and postoperative effect evaluation (14). Furthermore, iterations of 2D technology not only lack 3D anatomic detail but also expose operators and patients to relatively high radiation doses (15). Compared with 2-DSA, 3D-RV for the diagnosis and treatment of the iliac vein has the following advantages. First, 3D-RV with a curved planar reformat with a vessel cross-section is sufficient for diagnosing NIVL. This method applies the two-dimensional area ratio to assess the iliac vein stenosis rate, and the principle is the same as IVUS. Theoretically, 3D-RV can be localized and qualitatively and quantitatively evaluate the iliac vein compression, fully assess the extent and scope of the iliac vein stenosis, and improve the NIVL detection rate. Second, it can understand the internal and external iliac vein anatomy and fully display the arterial pressure traces, lumbar disc herniation, and endovascular conditions to provide direct evidence for the etiology of NIVL. Third, it can display collateral circulation, evaluate hemodynamic changes, and provide the hydrodynamic basis for NIVL treatment. Four, intraoperative fusion navigation was established by the 3D reality to display iliac vein bifurcation and lesion area, guide accurate stent implantation, and ensure the long-term patency of the stent (16). 3D-RV also can provide an optimal working angle while combining hemodynamics as a reference for subsequent treatment.

Presently, 3D-RV is widely applied in cerebrovascular angiography, and its advantage is that it can dynamically display the visualized vascular structures and walking. In the current study, the application of 3D-RV to the iliac vein compression lesions can better reflect the iliac vein compression and fluid dynamic information. 2-DSA requires multiple imaging to identify iliac vein compression from both AP and lateral projection imaging sites, causing additional exposure risks, whereas 3D-RV can be rotated from multiple angles to visually assess the extent and area of iliac vein lesions in dynamic imaging. With only one injection of contrast, the procedure time, X-ray dose, and contrast agent dosage were less than 2-DSA.

Iliac vein compression causes venous reflux disturbance; therefore, effective collateral circulation must be formed to compensate for the aggravation of stenosis (17). Contralateral iliac vein imaging is the most direct imaging manifestation of effective communication of pelvic collateral circulation, and it is also the main hemodynamic manifestation reflecting the iliac vein compression (18). In the current study, the increased proportion of patients with collateral vein development corresponds to the aggravation of iliac vein stenosis. Meanwhile, iliac vein compression was more severe in patients with pelvic c collateral and contralateral iliac vein imaging. Left common iliac vein indentations are mainly associated with the right iliac artery and posterior vertebral body compression. The left external iliac vein indentation may be that passing between the left internal and external iliac artery. External iliac vein stenosis is due to double artery pump-like compression, and external iliac vein indentations are another imaging indication of the severity of iliac vein compression.

This study has several limitations. Due to the relatively small sample size, there was a risk of type II statistical error. As a control arm, IVUS should be preferred over 2-D venography. However, IVUS-related data was not acquired due to the lack of the device, hence 2-D venography was selected as the control arm in this study. Finally, this study investigated the diagnostic value of three-dimensional rotary venography for non-thrombotic iliac vein lesions; therefore, the scope of follow-up after treatment is limited.

## Conclusion

In conclusion, 3D-RV can display dynamic stereo image information of NIVL, augmenting the information obtained from traditional 2-DSA. Contralateral iliac vein imaging, pelvic collateral vein imaging, and external iliac vein indentation can be used to evaluate the severity of iliac vein compression to some extent.

## Data availability statement

The original contributions presented in the study are included in the article/supplementary material, further inquiries can be directed to the corresponding author.

## Ethics statement

This study was approved by Binzhou Medical University Hospital. Written informed consent was obtained from all participants for their participation in this study.

## Author contributions

YX, WW, and XW: research idea, study design, and writing manuscript. YX, JW, and YC: sample and data acquisition. GC, XH, and YS: sample analysis. YS and WW: statistical analysis. All authors contributed to the article and approved the submitted version.
